# Event-Centered Data Segmentation in Accelerometer-Based Fall Detection Algorithms

**DOI:** 10.3390/s21134335

**Published:** 2021-06-24

**Authors:** Goran Šeketa, Lovro Pavlaković, Dominik Džaja, Igor Lacković, Ratko Magjarević

**Affiliations:** Faculty of Electrical Engineering and Computing, University of Zagreb, 10000 Zagreb, Croatia; goran.seketa@fer.hr (G.Š.); lovropavlakovic@gmail.com (L.P.); dominik.dzaja@fer.hr (D.D.); ratko.magjarevic@fer.hr (R.M.)

**Keywords:** fall detection, event-centered data segmentation, wearable sensors, accelerometer, window duration

## Abstract

Automatic fall detection systems ensure that elderly people get prompt assistance after experiencing a fall. Fall detection systems based on accelerometer measurements are widely used because of their portability and low cost. However, the ability of these systems to differentiate falls from Activities of Daily Living (ADL) is still not acceptable for everyday usage at a large scale. More work is still needed to raise the performance of these systems. In our research, we explored an essential but often neglected part of accelerometer-based fall detection systems—data segmentation. The aim of our work was to explore how different configurations of windows for data segmentation affect detection accuracy of a fall detection system and to find the best-performing configuration. For this purpose, we designed a testing environment for fall detection based on a Support Vector Machine (SVM) classifier and evaluated the influence of the number and duration of segmentation windows on the overall detection accuracy. Thereby, an event-centered approach for data segmentation was used, where windows are set relative to a potential fall event detected in the input data. Fall and ADL data records from three publicly available datasets were utilized for the test. We found that a configuration of three sequential windows (pre-impact, impact, and post-impact) provided the highest detection accuracy on all three datasets. The best results were obtained when either a 0.5 s or a 1 s long impact window was used, combined with pre- and post-impact windows of 3.5 s or 3.75 s.

## 1. Introduction

Falls among the elderly population are a major public health problem. Statistics from the World Health Organization (WHO) indicate that around 30% of adults over 65 years of age experience at least one fall per year [1]. Falls are one of the main causes of death in the elderly population [2]. Non-fatal falls also pose a problem because they leave a negative impact on both the physical and psychological health of elderly persons.

Negative consequences of a fall event can be reduced by shortening the time interval during which a person remains involuntary on the ground after the fall [3]. For this purpose, automatic fall detection systems can be used. Although fall detection systems are unable to prevent falls from happening, they can ensure that immediate assistance is provided to the faller by automatically detecting fall events and sending alarms to health professionals or caregivers. Because the faller might be unable to activate an alarm or search for help, it is important that such fall detection systems are automated [4].

Based on the sensor type used to detect falls, automatic fall detection systems are categorized as wearable or non-wearable. Wearable systems are placed on a person’s body with sensors that can track motion and gestures. On the other hand, non-wearable systems use sensors placed in a person’s environment, such as optical sensors, cameras, and floor sensors, to detect a fall. A review of different fall detection approaches can be found in [5]. A multitude of researchers have focused on wearable systems equipped with accelerometer sensors as they offer several advantages in terms of cost, power efficiency, ease of use, and portability [6]. Our work is also based on wearable accelerometer-based fall detection system records.

The process of accelerometer-based fall detection comprises three stages: data segmentation, feature extraction, and classification. The main goal of the data segmentation stage is to divide a continuous stream of data acquired from the accelerometer into segments, because features for classification can be extracted only from data segments of finite duration. Features are extracted from those data segments and passed to a classifier to discriminate whether the segment contains data from a fall event or from a regular Activity of Daily Living (ADL).

Different features [7] and classification techniques [8] have been explored for use in fall detection systems. In our previous work, we analyzed the performance of fall detection systems using different classification techniques: threshold-based classification [9,10] and different Machine Learning (ML) classifiers [11]. However, only a few research publications so far have focused on the data segmentation stage, although it significantly affects the systems performance in terms of power efficiency and detection accuracy [12,13].

Two approaches for data segmentation are used in fall detection research. In the first approach, a sliding window of a fixed duration (with or without overlap) is applied to the input data stream. The sliding window duration defines boundaries of a data segment from which further feature extraction and classification is performed. A description of fall detection systems that utilize sliding windows for data segmentation can be found in [14,15,16,17]. In the second approach, a trigger is initially set to detect a potential fall event in an input data stream by searching for acceleration peaks above a predefined threshold value. When a potential fall event is detected, one or multiple window(s) placed around this event determine data segments for further feature extraction and classification. Because a sudden change in acceleration is sensed in most falls at the moment a person hits the ground, with this approach, features are extracted from data segments centered around that potential impact point. This type of segmentation is thus called event-centered data segmentation.

Studies have shown advantages of using event-centered data segmentation over sliding windows. In [18], the performance of event-centered data segmentation with one window around a potential fall event was compared to sliding window segmentation for different window sizes. They found that the event-centered data segmentations performed slightly better than the sliding window based data segmentation. Putra et al. [19] proposed a fall detection system based on event-centered data segmentation with three windows. To evaluate the performance of the proposed system, they also measured performance when two different sliding window segmentation methods were used (with and without overlapping windows). They found that the event-centered segmentation outperformed sliding window segmentation while significantly reducing the computational cost.

The same as in [18], researchers in [20,21,22] also employed a single window for event-centered data segmentation. Another approach was used in [19,23,24,25,26], where data segmentation was based on multiple windows. Using more than one segmentation window is justified by an idea that in spite of the variable and irregular nature and typology of falls, they can be decomposed as a sequence of typical “stages” or phases. With this approach, the intention is to align data segmentation windows with different fall phases and thus extract more specific features for use in the classification stage.

Although event-centered data segmentation is regularly implemented in fall detection systems, no research so far has explored how different configurations of data segmentation windows affect the performance of the system in terms of detection accuracy. The aim of this paper is to fill this gap by comparing fall detection classifier performance in the case of implementation of either one, two, or three windows for event-centered data segmentation and to propose the optimal duration for each of these windows.

The remainder of the paper is organized as follows: Section 2 describes the methodology, followed by results in Section 3. A discussion of the results is provided in Section 4. Finally, Section 5 concludes the paper and points out the important practical implications of this study.

## 2. Materials and Methods

**Figure 1:**
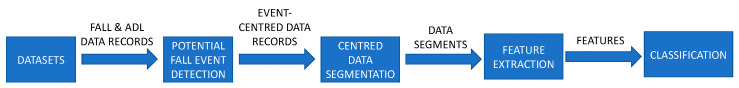
Architecture of the implemented testing environment for fall detection.

In our research, we implemented a fall detection testing environment, as shown in Figure 1. We used three publicly available datasets with fall and ADL records gathered from young participants in a controlled environment while wearing an acceleration sensor attached to the waist. Consequently, each record from the datasets contains only one fall or ADL activity. We first take fall and ADL data records from selected datasets and search for potential fall events. For each detected potential fall event, a part of the record before and after the event is extracted. Data segmentation with different window configurations is then applied to this event-centered data record. From there, data segments are obtained, and a set of features is calculated for each segment. Finally, a classifier is used to distinguish fall from ADL events, and its performance in terms of detection accuracy is evaluated. These steps are described in more detail in the following sections. For all the calculation and analyses in this study we used Matlab R2020b.

### 2.1. Fall Model

Although falls are diverse in etiologies (causes), circumstances, characteristics, and clinical consequences, a fall can generally be defined as “an unexpected event in which the person comes to rest on the ground, floor, or lower level” [27]. For research purposes, falls are usually described as a sequence of multiple phases. Models with different numbers of phases have been proposed [28,29,30], but a model with three phases is most widely accepted.

A fall starts when a person loses balance and starts an uncontrolled descent towards the ground that can no longer be recovered by protective strategies. The period between the start of the fall and the body impact on a lower surface is often called the pre-impact or falling phase. During this phase, the acceleration towards the ground is in most cases less than 9.81 $\frac{m}{s^{2}}$ (1 g), but it can be influenced by balance recovery attempts such as stepping strategies or grabbing on to other objects. The total duration of this phase depends on the circumstances and the balance recovery strategies employed by the faller.

The moment when a person hits the ground or some other lower surface for the first time is considered the beginning point of the impact phase. This moment usually causes an abrupt change of the acceleration direction. The magnitude of acceleration change depends on falling dynamics and type of ground surface.

At the end of the impact phase, the person is lying or sitting on the ground or other lower surface. This phase is called the rest phase. If the person is unable to move due to the fall, no significant changes in acceleration magnitude can be observed in this phase. However, this is not the case if the person makes attempts to recover from the fall.

**Figure 2:**
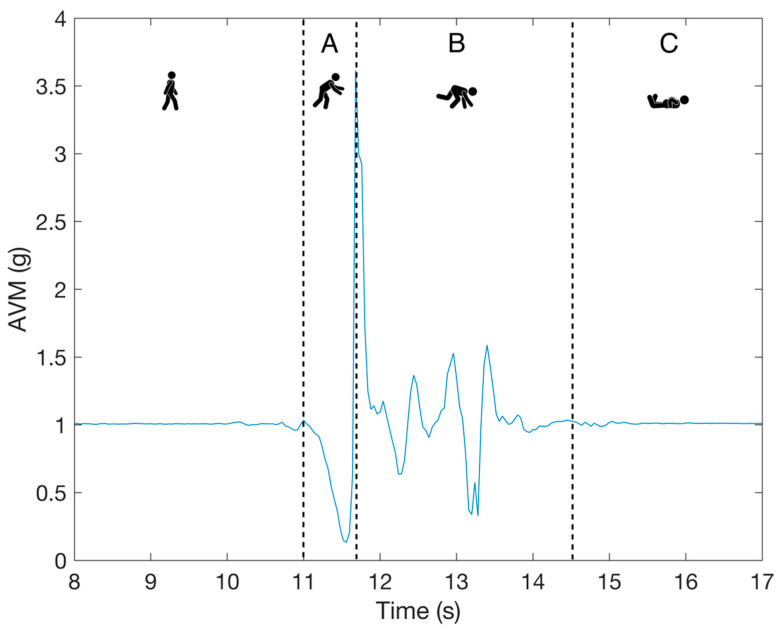
An example of a fall signal with three phases: A is the pre-fall phase, B is the impact phase, and C is the rest phase.

An example of a fall event measured with a tri-axial accelerometer is shown in Figure 2. The figure displays measurements from three accelerometer axes combined into a single value called *Acceleration Vector Magnitude* (*AVM*). *AVM* is calculated according to Equation (1):

(1) $$AVM\left[i\right]=\sqrt{{\left(a_{x}\left[i\right]\right)}^{2}+{\left(a_{y}\left[i\right]\right)}^{2}+{\left(a_{z}\left[i\right]\right)}^{2}},\;$$

where i is the current data sample and $a_{x}$, $a_{y}$, and $a_{z}$ represent, respectively, the acceleration signals in the *x*, *y*, and *z* axes of the sensor. Accelerations and the *AVM* value are thereby expressed in g units (1 g = 9.81 $\frac{m}{s^{2}}$).

### 2.2. Datasets

Three publicly available datasets that contain acceleration measurements of falls and ADL were used in this research: ErciyesUni [17], FallAllD [31], and SisFall [32]. For all three datasets, young subjects performed a variety of simulated falls and ADL in a controlled environment while wearing accelerometer sensors attached to different body parts. In this study, we used only records from the waist sensor because this position was used by all three datasets.

Fall detection systems are mainly intended for use by elderly populations, but recording unintentional falls from elderly people in real life is a complex task. Because real-life falls are rare events, recording them is both time consuming and costly [33]. The FARSEEING consortium, consisting of 10 partners from 5 EU countries, succeeded in recording 300 real-world fall events with inertial sensors over 4 years (from January 2012 to December 2015) [34]. From this collaborative project, a subset of 20 falls is publicly available. So far, no open datasets are available that contain a significant number of real life elderly falls. Therefore, the majority of studies still use data from simulated falls of young healthy subjects recorded in a safe environment [35].

A separate record was created for each performed ADL or fall. In this way, each record stored in the dataset contains only one type of fall or ADL and is uniquely labeled with an anonymized subject identifier, activity type (e.g., frontal fall to the knees, ADL sitting down on a chair, etc.), and trial number. Besides these common characteristics, datasets were created by different research groups and with distinct experimental protocols.

The ErciyesUni dataset contains sensor measurements from 17 subjects (age: 19–27, weight: 47–92 kg, height: 157–184 cm), acquired while they performed a set of scripted ADL and simulated falls. In total, the dataset contains 1360 ADL and 1700 fall records. Every subject performed 16 types of ADL and 20 different types of falls with 5 repetitions while wearing 6 sensing units (Xsens MTw Motion Tracking Kit, Xsens, Enschede, The Netherlands). Those sensing units measured accelerations of different body parts by accelerometers (measurement range ±16 g, sampling frequency 25 Hz). They were worn by the subjects attached to different body parts: head, chest, waist, wrist, tight, and ankle. In this work, we used data from the waist worn sensing unit in order to process signals from the same sensor position because signals from that position are present in all three selected datasets.

FallAllD is a dataset of falls and ADL records simulated by 15 volunteering participants. Each participant performed 35 types of simulated falls and 44 types of ADL. The average age, height, and weight of participants were 32 years, 171 cm, and 67 kg, respectively. The participants were asked to wear a sensing unit around their neck and wrist, and attached to the waist while performing predefined movements. Each sensing unit was equipped with four sensors: an accelerometer, a gyroscope, a magnetometer, and a barometer. For our study, we used acceleration data from the sensing unit attached to the waist. The measurement range of the employed accelerometer was ± 8 g and the sampling frequency was 238 Hz.

The SisFall dataset was acquired by SISTEMIC group (University of Antioquia, Medellin, Colombia). This dataset contains measurements from a group of 15 elderly subjects and a group of 23 young adults. For this study, we used fall and ADL data collected from young subjects only (age 25.0 ± 8.6 years, height 165.7 ± 9.3 cm, weight 57.7 ± 15.5 kg) because the elderly group did not perform simulated falls. Acceleration and angular velocity measurements were acquired with an inertial sensor unit (Shimmer sensing, Ireland) while subjects wore the sensor attached to their waist and performed a set of 15 different types of falls and 19 types of ADL. In total, 2707 ADL data records and 1798 fall data records were acquired. Acceleration was measured with two accelerometers embedded on the Shimmer sensing unit: ADXL345 (measurements range ±16 g) and MMA851Q (measurements range ±8 g). A sampling frequency of 200 Hz was employed for acceleration measurements. For this study, we selected data from the accelerometer ADXL345 due to the larger measurement range.

**Figure 3:**
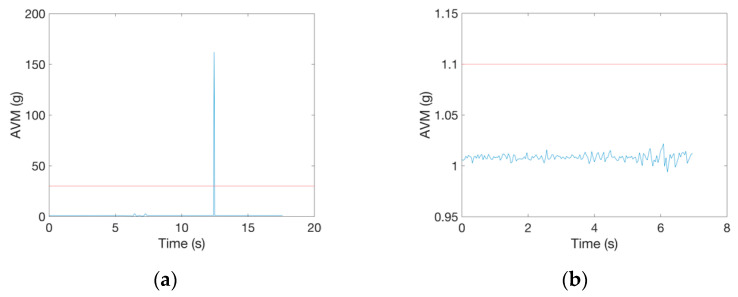
Examples of fall records in the ErciyesUni dataset excluded from further analysis due to: (**a**) the maximal *AVM* peak value larger than 30 g and (**b**) the maximal *AVM* peak value lower than 1.1 g.

From all three datasets, we excluded data records that contained physically not interpretable data and were therefore most likely caused by a measurement/sensor error. All falls and ADL data with the maximal value of *AVM* larger than 30 g and all falls data with maximal peak value lower than 1.1 g were excluded from further analysis. The constraint of 30 g was chosen because the accelerometer measurement range in the employed datasets was ±16 g (SisFall and ErciyesUni) and ±8 g (FallAllD). So, even if acceleration values from the top of these ranges (16 g and 8 g) would have been recorded during a fall or ADL in all three axes, the value of *AVM* calculated according to Equation (1) would be less than 30 g. We chose the value of 1.1 g to discard all fall records in which potentially no fall was recorded because this value is just slightly larger than *AVM* recorded during rest (1 g) and at the same time far enough from the minimal recorded acceleration peak of 1.6 g, which was found in a study on real world falls [36]. We discarded 7 falls and 3 ADL from the ErciyesUni dataset due to these criteria. Example of such signals are shown in Figure 3.

Additionally, to ensure that the time span of the records was long enough for data segmentation, only fall records with the largest *AVM* peak recorded more than 5 s before the end of the signal record were taken into consideration. In total, 49 fall records from the SisFall dataset did not satisfy this criteria and they were not used in this research. From the FallAllD dataset, all records satisfied the criteria.

### 2.3. Potential Fall Event Detection

The main goal of potential fall event detection is to detect changes in acceleration that might come from a fall. By detecting a potential fall event, only a preselection of data is made; all potential fall events are further processed for the decision as to whether they actually come from a fall or from some fall-like ADL. A potential fall event detection algorithm has to be simple, fast, and able to accurately identify all fall events while rejecting ADL as much as possible. This way, the computational cost of the system is reduced because the more complex ML based algorithm is utilized only for fall-like events while most of the ADL is already rejected. Another benefit of this approach is that it provides a center point for data segmentation based on extracting features from specific fall phases.

Potential fall event detection is based on detecting a sudden and large increase of acceleration magnitude in the input signal that can be observed during fall impact [26]. The impact is the most prominent part of a fall signal measured with accelerometer sensors. In the impact phase, when the faller hits the ground or some other lower level surface, an abrupt change of the direction in acceleration signals occurs. This change is due to the breaking acceleration opposite to the initial fall direction.

**Figure 4:**
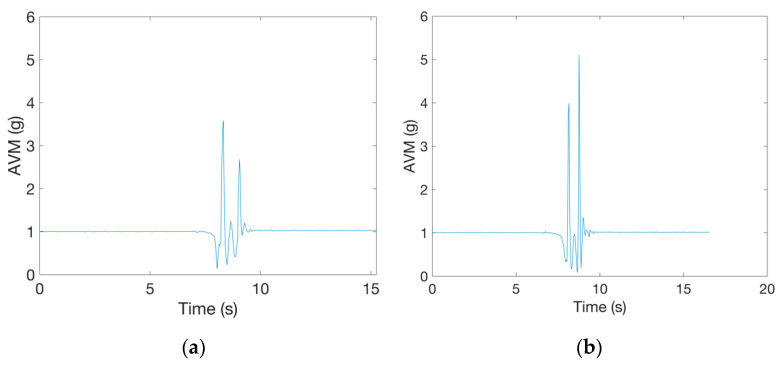
Examples of the same fall type from the ErciyesUni dataset simulated by two subjects: (**a**) the impact to the knees produces larger *AVM* peak than the impact of the body to the ground; (**b**) the *AVM* peak is larger when the upper part of the body impacts the ground.

During a fall, multiple high acceleration peaks may be produced as a result of the protective actions the person performs to avoid or reduce the consequences of the impact. Examples are protective arm movements, falling to knees to break the fall in two parts, or holding on to objects to slow down the fall [28]. During a fall, a person can also hit other objects. The presence of multiple acceleration peaks in a fall signal may cause detection of multiple possible fall events, thus making the alignment of fall phases more difficult. Examples of two acceleration signals measured during a broken fall to the knees from the ErciyesUni dataset are presented in Figure 4. The figure shows records of the same type of fall simulated by two subjects. In these falls, subjects first fell to their knees and then continued to fall until their chest touched the ground. In the first example, the maximal *AVM* peak occurred during the initial impact to the knees, while in the second example, a larger *AVM* peak, was measured when the upper part of the body impacted with ground.

Methods for event detection that avoid multiple-peak problems have been proposed in the literature [19,20,37,38]. They are all based on similar reasoning. Because falling down is a single event that happens suddenly, a fall-like event should not have traits of repetitiveness and can be characterized as an acceleration peak higher than a predefined threshold followed by a period without peaks larger than the threshold. We implemented a potential fall event detector following this idea.

Firstly, we calculated the *AVM* for each data sample. If the *AVM* value exceeded a fixed threshold, we analyzed further data in the period after the sample that was larger than the threshold. Then, if the *AVM* value of all data points in that period were lower than the threshold, a potential fall event was detected.

**Figure 5:**
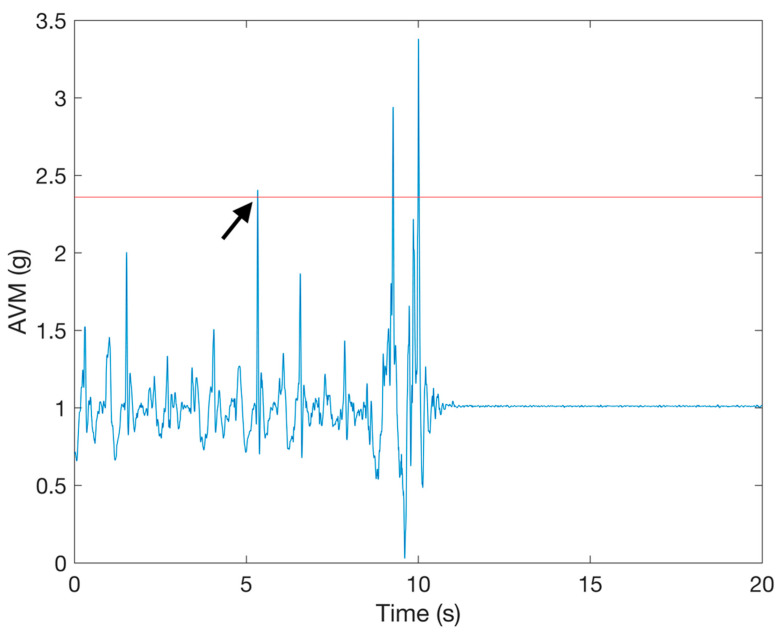
An example of a fall record from the FallAllD dataset that contains an ADL before the fall. The arrow indicates the point in the signal where the ADL causes detection of a false potential fall event.

We had to choose the duration of the period in which we looked for further peaks after the *AVM* acceleration peak. A similar method for event detection was used in a previous study [37] and yielded good results with a time period of 2.5 s, so we chose 2.5 s as the time period in which we look for further peaks after the acceleration peak. In order to find the best thresholds for each dataset, we tested the method of potential fall detection with a range of threshold values for all fall records. The threshold values were varied from 0 to 5 g with a step of 0.005 g. Only those potential fall events detected after the largest *AVM* peak in fall records were labeled as true fall events. This criterion was set because the FallAllD and SisFall datasets contain ADL activities in fall records prior to the fall event. An example of a fall record where a potential fall event is detected during ADL before the fall is shown in Figure 5. By using that additional criterion, we ensure that a potential fall event detected in the ADL part of a fall record is correctly labelled as a false fall event.

We selected the largest threshold for which in all fall records at least one true positive event was detected. Choosing the largest threshold minimizes the number of false alarms with ADL data. The thresholds chosen for each dataset are as follows:

- ErciyesUni: 1.330 g;
- FallAllD: 2.360 g;
- SisFall: 1.775 g.

Using this method, we detected potential fall events from ADL and fall records. We then extracted data of each potential fall event containing 4 s of data both before and after the event into a new record (event-centered data record). All event-centered data records that were taken from ADL dataset records were labeled as ADL. Additionally, all parts of the records from fall signals occurring before the largest peak were labeled as ADL (because they were triggered by an activity before the fall, as discussed previously). Only event-centered data records from falls that are detected after the largest *AVM* peak were labeled as falls.

The maximal period of 4 s was chosen for data extraction because of the limitations in data from the public datasets used in this study. Namely, those datasets provide records of falls and ADL with limited duration. For this research, it is beneficial to have as much data as possible available before and after each potential fall event in both fall and ADL records. The duration of 4 s was chosen to provide a fair amount of time for data segmentation analysis while preventing too many ADL and fall records being discarded due to a lack of data (to short signal records) being available for analysis.

The final number of event-centered data records chosen for further processing is listed in Table 1. For comparison, Table 1 also contains the number of records that would be available if all potential fall events were used, neglecting the criterion of minimal time for analysis (limit 0 s). The number of data records that would be available with a time limit of 5 s is also given. Raising the limit from 4 s to 5 s would lead to a significant reduction of available data from the SisFall dataset and therefore we found it not acceptable in our study.

### 2.4. Event-Centered Data Segmentation

When a potential fall event is detected, a single window or multiple windows before and after the event are used to define boundaries of the data segments from which features for classification are calculated. So defined data segments should contain characteristics of the entire fall or of specific fall phases. There are, however, multiple window configurations that can be used to extract these data segments. The aim of this research was to explore how these window configurations in the data segmentation stage influence performance of the fall detection system and to find the best performing one. Performance was thereby measured by the ability of the system to correctly detect falls when they really occurred while avoiding raising false alarms for ADL.

**Figure 6:**
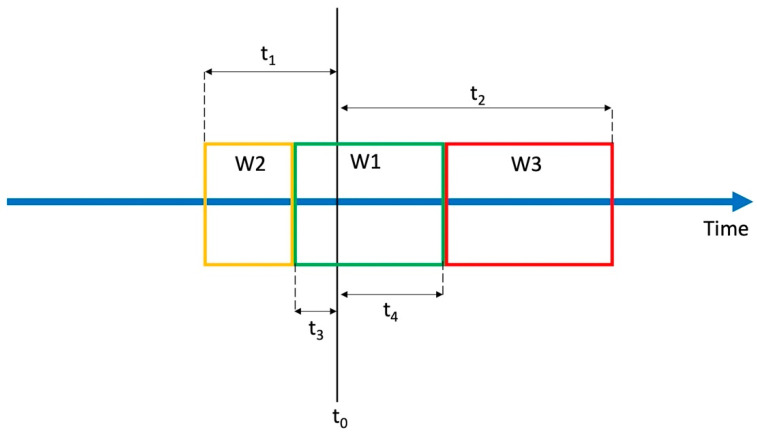
The model for testing different window configurations in event-centered data segmentation; $t_{0}$ marks the occurrence of a potential fall event. Parameters $t_{1–4}$ determine the time between $t_{0}$ and the beginning or the end of a window.

We used the model presented in Figure 6 to create different configurations of windows with varying durations.

The model consists of three sequential and coupled windows labeled W1, W2, and W3, and 4 timing parameters labeled $t_{1}$, $t_{2}$, $t_{3}$, and $t_{4}$. Each window spans a segment of input data. Parameters $t_{1–4}$ determine the time between the beginning or the end of a window and the detected potential fall event at time, $t_{0}$. The impact window, W1, spans between $\left(t_{0}-t_{3}\right)$ and $\left(t_{0}+t_{4}\right)$, the pre-impact window, W2, spans between $\left(t_{0}-t_{3}\right)$ and $\left(t_{0}-t_{1}\right)$, and finally the post-impact window, W3, spans between ($t_{0}+t_{4})$ and $\left(t_{0}+t_{2}\right)$. By varying the criteria for parameter inclusion and duration, different configurations of windows can be created, as shown in Table 2.

Parameters $t_{1}$ and $t_{2}$ were varied from 0 to 4 s, with a step size of 0.5 s. For each value of $t_{1}$, parameter $t_{3}$ was changed from 0 to $t_{1}$, with steps of 0.25 s. Similarly, $t_{4}$ was varied in range from 0 to $t_{2}$ for each value of $t_{2}$ in steps of 0.25 s. All possible combinations of parameter values $t_{1–;4}$ were tested. The maximal window duration is limited to 4 s due to the available duration of data in the event-centered data records. This issue was discussed in Section 2.3.

### 2.5. Feature Extraction

We calculated a set of features from each data segment provided by event-centered data segmentation. Features should gather distinctive parameters that are used by the classifier to differentiate between falls and ADL. The set of features we chose for this work is commonly used in fall detection research [18]. In total, eight features were calculated for each segment from tri-axial acceleration data ($a_{x}$, $a_{y}$, $a_{z}$) or *AVM* according to Equations (2)–(9). All features and variables used to calculate them were expressed in g units (1 g = 9.81 $\frac{m}{s^{2}}$).

(2) $$\bar{AVM}=\frac{1}{N}\overset{N}{\underset{i=1}{\sum }}AVM\left[i\right]$$

(3) $$AVM_{max}=\underset{i=1,2,\ldots N}{\max}AVM\left[i\right]$$

(4) $$AVM_{min}=\underset{i=1,2,\ldots N}{\min}AVM\left[i\right]$$

(5) $$AVM_{range}=AVM_{max}-AVM_{min}$$

(6) $$s_{N}=\sqrt{\frac{1}{N}\overset{N}{\underset{i=1}{\sum }}{\left(AVM\left[i\right]-\bar{AVM}\right)}^{2}}$$

(7) $$SMA=\overset{N}{\underset{i=1}{\sum }}\left(\left|a_{x}\left[i\right]\right|+\left|a_{y}\left[i\right]\right|+\left|a_{z}\left[i\right]\right|\right)$$

(8) $$AAMV=\frac{1}{N}\overset{N}{\underset{i=1}{\sum }}\left|AVM\left[i+1\right]-AVM\left[i\right]\right|$$

(9) $$AVM_{rms}=\sqrt{\overset{N}{\underset{i=1}{\sum }}\left(a_{x}{\left[i\right]}^{2}+a_{y}{\left[i\right]}^{2}+a_{z}{\left[i\right]}^{2}\right)}$$

where *N* is the number of samples in a data record, $\bar{AVM}$ is the mean, and $s_{N}$ is the standard deviation of all AVM samples in a record. The maximal and the minimal values and the difference between the maximal and minimal values (range) of AVM samples in a record are given by Equations (3)–(5), respectively. The *Summed Magnitude Area* (*SMA*) is the sum of the absolute values of the acceleration components in all three axes in a record, and it is calculated according to Equation (7). The *Average Absolute Acceleration Magnitude* (*AAMV*) is calculated according to Equation (8) as a difference between two consecutive *AVM* samples. Finally, $AVM_{rms}$ is calculated as a square root of the sum of squared acceleration values in all three axes (Equation (9)).

Although orientation of the sensor can be estimated from tri-axial accelerometer data, features based on orientation were not used in this study. Estimation of orientation assumes a known orientation of the accelerometer sensor axes with respect to the wearer’s body. In a real life application of a fall detection system, this would require a user to always wear the sensor at a predefined orientation. This reduces the usability of the system and therefore we preferred solutions that do not depend on posture information.

### 2.6. Classification and Performance Evaluation

For the training of the machine learning algorithm, segments from each event-centered data record were individually labeled as either a fall or an ADL.

We used the fitcsvm function from MATLAB’s Statistics and Machine Learning Toolbox to implement an SVM classifier. Previous works in the field of fall detection systems have shown good performance results for the SVM classifier [11,39]. Basically, SVM tries to find the best hyperplane that maximizes the margins between each of the classes. Several hyperparameters affect the classification result with the SVM classifier: *C*, *gamma*, and *kernel*. We standardized the features and used the radial basis function *kernel*. Hyperparameter value *C* for the SVM classifier was set to 1. The parameter *gamma* was automatically set to an appropriate value by the software using a heuristic procedure.

We employed five-fold cross validation to evaluate the performance of the classifier. All data records were randomly partitioned into five portions. Then four portions were utilized as training data and one portion as testing data. This was repeated five times until each portion was used as the testing set. Averaged test results over all iterations were taken.

The following metrics were used to evaluate the test results:

(10) $$F_{score}=\frac{2TP}{2TP+FP+FN}$$

(11) $$AMR_{type}=\frac{FN_{type}}{FN+TP}$$

(12) $$AFPR_{type}=\frac{FP_{type}}{FP+TN}$$

where *TP*, *FP*, *FN*, *TN,* $FN_{type}$, and $FP_{type}$ are defined as follows:

- *TP* = number of all true positive records; a data record is determined as a *TP* if it is labeled and detected as a fall;
- *FP* = number of all false positive records; a data record is determined as an *FP* if it is labeled as an ADL and detected as a fall;
- *FN* = number of all false negative records; a data record is determined as an *FN* if it is labeled as a fall and detected as an ADL;
- *TN* = number of all true negative records; a data record is determined as a *TN* if it is labeled as an ADL and detected as an ADL;
- $FN_{type}$= number of *FN* records for a particular type of fall;
- $FP_{type}$= number of *FP* records for a particular type of ADL.

$F_{score}$ is a harmonic mean of sensitivity and precision and is often used as a single standard measure for evaluation of fall detection systems [33,40,41]. $AMR_{type}$ (from Activity Miss Rate) and $AFPR_{type}$ (from Activity False Positive Rate) express percentage of records from a particular ADL or fall type that are misclassified. Thereby, $AMR_{type}$ is the percentage of falls of one type that are not detected and $AFPR_{type}$ is the percentage of ADL of a single type that are detected as falls.

## 3. Results

The performance of the SVM classifier was evaluated with different configurations of windows in the data segmentation process. A total of 6560 classification results per dataset were obtained for all combinations of window parameter values $t_{1–4}$.

The highest classifier performances achieved when using one, two, and three windows for data segmentation are listed in Table 3. Firstly, subsets of all results relevant to each window combination were extracted. The criteria for parameters $t_{1–4}$ given in Table 2 were used to obtain each subset. For example, to acquire the subset of all results when one window is used, all combinations were selected for which $t_{1}$ is equal to $t_{3}$ and $t_{2}$ is equal to $t_{4}$. This subset then contains results of classification when window W1 with different durations, defined by $t_{3}$ and $t_{4}$, are used. The highest classifier performance reported in Table 3 is then simply the maximal value of the $F_{score}$ achieved in the subset.

The results show that the highest classification performance is achieved when all three windows, W1–W3, were used for data segmentation. This combination has the highest maximal achieved $F_{score}$ in all three datasets (99.7% in the ErciyesUni dataset, 96.1% in the FallAllD dataset, and 98.4% in the SisFall dataset). Generally, the lowest scores in all three datasets are obtained when only one window is used.

The values of parameters $t_{1–4}$, for which maximal $F_{score}$ are achieved with data segmentation based on three windows, are listed in Table 4. Because the results differ between datasets, we explored whether a range of parameters can be found that performs well in all datasets.

**Figure 7:**
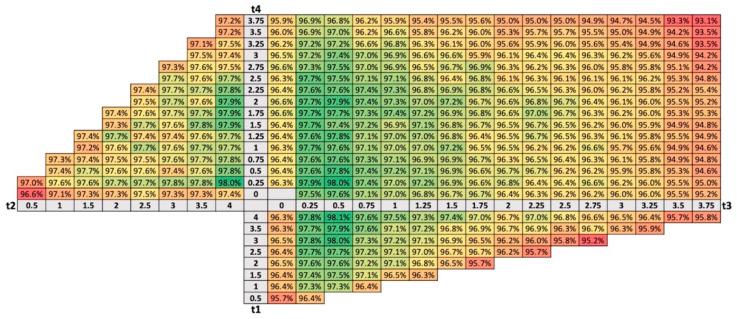
The average of maximal $F_{score}$ from all datasets for each pair of parameters ($t_{3}$,$\;t_{4}$), ($t_{2}$, $t_{4}$), ($t_{1}$, $t_{3}$). Scores are color coded where darker green is showing higher scores while darker red is showing lower scores.

The size of each window for data segmentation is determined by a pair of parameters: W1 ($t_{3}$,$\;t_{4}$), W2 ($t_{2}$, $t_{4}$), W3 ($t_{1}$, $t_{3}$). The maximal $F_{score}$ for all tested combinations of parameter pairs were calculated for every dataset. An average of scores from all three datasets is shown in Figure 7. All scores are color coded to facilitate the analysis, where darker green is showing better scores while darker red is showing low values in scoring.

The best results in the three window-based data segmentation approaches were obtained when parameters $t_{1}$ and $t_{2}$ were set to the maximal value tested, 4 s, and when the $t_{3}$ and $t_{4}$ values were either 0.25 s or 0.5 s. Because these parameters define the duration of the segmentation windows, as stated in Section 2.4, we can express these results in terms of window duration. Thus, the best results were obtained for a shorter duration of the impact window, W1 (0.5 s or 1 s), and a longer duration of the pre-impact and post-impact windows, W2 and W3 (3.5 s or 3.75 s).

## 4. Discussion

In this study, we implemented a testing environment for a fall detection system in order to explore how the configuration of windows used in event-centered data segmentation affects the detection accuracy. Configurations of one to three windows with varying window durations were used at the data segmentation stage. Performance of an SVM classifier was evaluated with $F_{score}$ metrics for all configurations.

**Figure 8:**
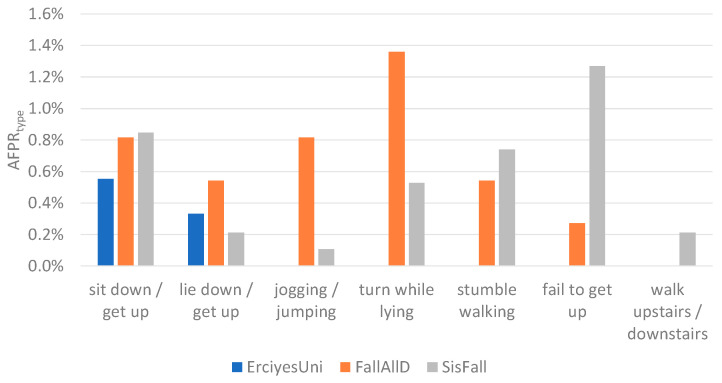
Percentage of all ADL misclassified as fall, grouped by ADL types **(**$AFPR_{type}$).

**Figure 9:**
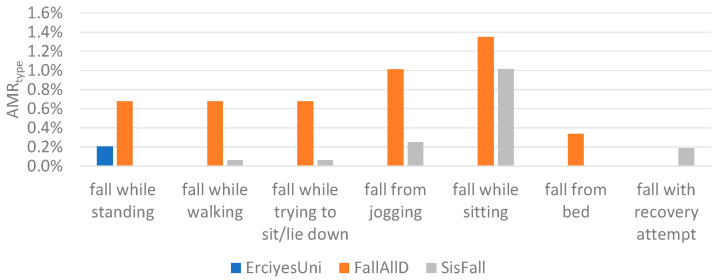
Percentage of all falls misclassified as ADL, grouped by fall types ($AMR_{type}$).

The results from Table 3 show that the highest $F_{score}$ is achieved when three windows are used for event-centered data segmentation. In the ErciyesUni dataset, the difference between the performances achieved with one, two, and three windows was small, and all of the scores were higher than 99%. In the FallAllD and SisFall datasets, the difference was more prominent and the highest achieved scores were lower than in the ErciyesUni dataset. Overall performances differ between datasets due to the heterogeneity of fall and ADL types present in each dataset. Figure 8 and Figure 9 show the $AMR_{type}$ and $AFPR_{type}$ of activities for the configuration where the highest $F_{score}$ is achieved with three windows for data segmentation. Similar activities were grouped together for a better overview. Some of the ADL types that caused false positive alarms in the FallAllD and SisFall dataset, such as turning in bed, failed attempt to get up from a chair, and walking up stairs, are not present in the ErciyesUni dataset. ErciyesUni does not contain any falls that follow an ADL (such as falls during walking, jogging, or sitting). The lack of ADL and fall types that are more difficult for classification in ErciyesUni may be the cause of the better scores achieved compared to the FallAllD and SisFall datasets. Heterogeneity of data between datasets has been the focus of some previous studies [2,35,42,43,44], which have shown that the type of activities contained in datasets for fall detection differs significantly. Moreover, difference between falls and ADL types contained in the datasets is one of the factors that explains the difference in the threshold values for potential fall event detection presented in Section 2.3.

Data segmentation approaches for fall detection have been explored in previous studies. In their study, Putra et al. [19] compared an event-centered data segmentation approach with sliding window segmentation. They found that event-centered data segmentation based on three windows outperformed segmentation with a single sliding window in terms of computational efficacy and detection accuracy. In [18], detection accuracy between a single window for event-centered data segmentation was compared to data segmentation with an overlapping sliding window. On the other hand, instead of comparing performance between the sliding window and the event-centered data segmentation approach, we focused on finding the best performing configuration of windows for the event-centered segmentation. To the best of our knowledge, our study is the first to compare the usage of one, two, and three windows in event-centered data segmentation.

Further, we analyzed the effect of window durations on the system’s performance when a configuration of three windows for data segmentation is used. Figure 7 shows the $F_{score}$ values achieved for parameter pairs that define each window: W1 ($t_{3}$,$\;t_{4}$), W2 ($t_{2}$, $t_{4}$), W3 ($t_{1}$, $t_{3}$). Thereby, the $F_{score}$ value represents the average of the highest scores from all three datasets used in the study. As shown in the figure, for parameter $t_{3}$, better scores are achieved when using lower values (0.25 s and 0.5 s). This is the parameter that defines the duration of window W1 in the period before a potential fall event and therefore incudes the impact peak. For parameter $t_{1}$, the best results were achieved with longer values (maximal tested value of 4 s). This means that longer durations of W2 that capture activity before the fall are favored (short $t_{3}$ and long $t_{1}$ values). As with $t_{1}$, longer values of $t_{2}$, close to 4 s, performed best. With parameter $t_{4}$, similar scores are achieved in a range of values lower that approximately 2.5 s. This parameter defines the amount of post-impact data included in window W1 as well as the starting point and duration of window W3. In one way, lower values of $t_{4}$ reduce the amount of post-impact data to interfere with impact focused window W1 and enable a longer duration of W2 for gathering data during rest after the fall. On the other hand, longer values of $t_{4}$ provide a time offset for the beginning of window W2 after the impact, so that less intermediate post-impact data is included in the rest analysis. The choice of the value of the parameter $t_{4}$ is therefore a compromise. Nevertheless, lower values for $t_{4}$ (less than 0.5 s) provided slightly better results.

To summarize, in our study, the best results were obtained when parameters $t_{1–4}$ were set to values listed in the first row of Table 5. Because these parameters define the duration of segmentation windows, as described in Section 2.4, we can also express our results in terms of window durations. Hence, when three sequential windows for event-centered data segmentation are used, we recommend a shorter duration of impact window W1 (0.5 s or 1 s) and longer durations of pre- and post-impact windows W2 and W3 (3.5 s or 3.75 s).

Event-centered data segmentation with three windows has been utilized in previous studies with different window durations. In [19], pre-impact, impact, and post-impact windows of 1 s were used around the impact peak. Hsieh et al. [24] presented an adaptive approach where the duration of the impact window depended on the amplitude of the largest acceleration peak on record. In [25], an impact window of 1.5 s before and a window of 0.5 s after the largest acceleration magnitude was taken. Two additional windows were then placed before and after the impact window. In Table 5, the durations of the windows used in the aforementioned studies are expressed with parameters $t_{1–4}$ for easier comparison to our results.

An analysis of the effect of the size of windows on fall detection accuracy was previously performed by [18]. They measured performance of a fall detection system with event-centered data segmentation using a single window of varying duration. The best results were achieved when they used a window of 3 s centered around the potential fall event. On the contrary, the focus of our study was in finding the best performing durations of each of three windows, because they showed better detection accuracy compared to using a single window.

For the purpose of this study, we employed three publicly available datasets with acceleration data from young subjects performing simulated falls and ADL. That is a limitation of our research because fall detection systems are aimed at assisting the elderly population. Some of the research indicates that the patterns of falls experienced by elderly people is similar to simulated falls from young subjects [45,46]. Acquiring data from elderly people that experience falls in real life situations is challenging, and only a few researchers have worked to acquire them [26,34,47,48,49]. Those acquired datasets are not publicly available.

Another limitation of this study is that we had to restrict our analysis of window durations to 4 s due to the length of data available in each fall or ADL record. The best results for window lengths of $t_{1}$ and $t_{2}$ were found to be at the maximal explored value of 4 s. In our future work, we plan to create a database of simulated falls and ADL activities with data records of sufficient duration to analyze longer ranges of window sizes.

Based on our findings for the parameter $t_{4}$ value, we plan to explore a more complex model of windows for data segmentation. With that model, two additional parameters will be introduced so that configurations with overlapping or separated windows can be investigated as well.

## 5. Conclusions

Data segmentation is an important part of automatic fall detection systems because it affects the overall detection accuracy. In this work, we explored different window configurations that can be used with event-centered data segmentation. A fall detection system based on an SVM classifier was built, and three publicly available datasets with fall and ADL records were used for the test. We compared the fall detection classifier’s performance in the case of implementation with either one, two, or three windows for event-centered data segmentation. We found that using three windows for data segmentation yields better fall detection performance than using one or two windows. Finally, we analyzed a range of window durations and found that the best results were obtained with a shorter duration of impact window, W1 (0.5 s or 1 s), and a longer durations of pre- and post-impact windows, W2 and W3 (3.5 s or 3.75 s). These findings can be used as a guideline for implementing event-centered data segmentation in fall detection systems.

## Figures and Tables

**Table 1:** The number of event-centered data records referred to the minimal time available before and after a potential fall event.

Dataset	Minimal Time Available (s)	Number of Fall Records	Number of ADL Records
ErciyesUni	0	1460	914
	4	1454	904
	5	1448	883
FallAllD	0	314	472
	4	301	368
	5	300	335
SisFall	0	1823	1308
	4	1649	945
	5	1433	667

**Table 2:** Criteria for parameters to form specific data segmentation window configurations for testing.

Number of Windows in Configuration	Window Configurations	Criteria for Parameters
1	W1	*t*_1_ = *t*_3_ AND *t*_2_ = *t*_4_
2	W1 & W2	*t*_2_ = *t*_4_ AND *t*_1_ > *t*_3_ AND (*t*_3_ > 0 OR *t*_4_ > 0)
	W1 & W3	*t*_1_ = *t*_3_ AND *t*_2_ > *t*_4_ AND (*t*_3_ > 0 OR *t*_4_ > 0)
	W2 & W3	*t*_3_ = 0 AND *t*_4_ = 0 AND *t*_1_ > 0 AND *t*_2_ > 0
3	W1 & W2 & W3	*t*_1_ > 0 AND *t*_2_ > 0 AND *t*_1_ > *t*_3_ AND *t*_2_ > *t*_4_ AND (*t*_3_ > 0 OR *t*_4_ > 0)

**Table 3:** The maximal $F_{score}$ achieved for each configuration of data segmentation window.

Number of Windows in Configuration	Window Configurations	$\mathrm{Max}\;F_{score}\;\mathrm{ErciyesUni}\;(\%)$	$\mathrm{Max}\;F_{score}\;\mathrm{FallAllD}\;(\%)$	$\mathrm{Max}\;F_{score}\;\mathrm{SisFall}\;(\%)$
1	W1	99.2	89.5	94.2
2	W1 & W2	99.5	94.0	97.3
	W1 & W3	99.6	93.0	97.1
	W2 & W3	99.3	92.5	96.5
3	W1 & W2 & W3	99.7	96.1	98.4

**Table 4:** Values of parameters $t_{1–4}$ for which the best performance is achieved for each dataset when three windows are used for segmentation.

Dataset	$t_{1}\;(s)$	$t_{2}\;(s)$	$t_{3}\;(s)$	$t_{4}\;(s)$
ErciyesUni	4	3.5	0.5	0.5
FallAllD	3	4	0.5	0.25
SisFall	4	3.5	0.5	0.25

**Table 5:** Duration of data segmentation windows used in previous research compared to the ranges recommended in this work.

Study	$t_{1}\;(s)$	$t_{2}\;(s)$	$t_{3}\;(s)$	$t_{4}\;(s)$
our study	4	4	0.25 or 0.5	0.25 or 0.5
Putra et al. [19]	1	2	0	1
Hsieh et al. [24]	0.3281	2.5	0.07815	0.0781 or 0.156 ^1^
Zurbuchen et al. [25]	rest of the record ^2^	rest of the record ^2^	1.5	0.25

^1^ depends on the event peak amplitude; longer duration is used for peak amplitudes < 6 g. ^2^ value of $t_{1}$ and $t_{2}$ depends on the duration of the record after the window around the event is formed.

## Data Availability

Not applicable.
